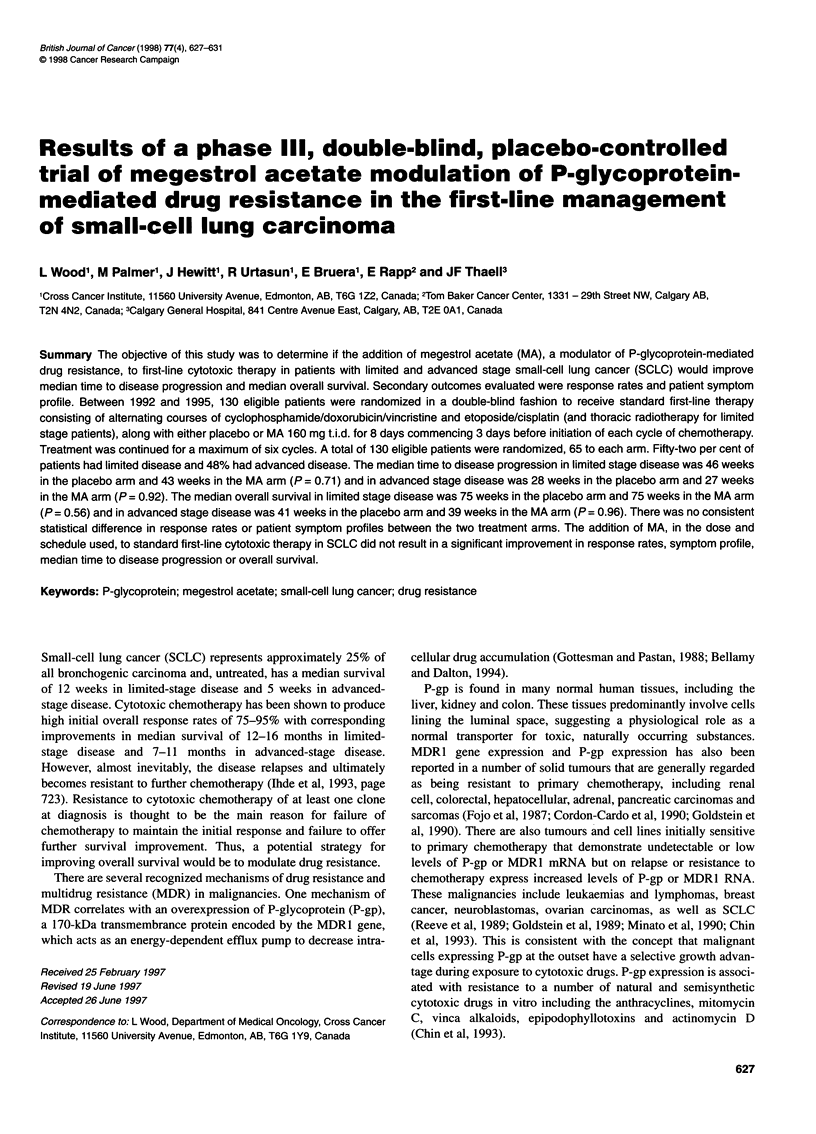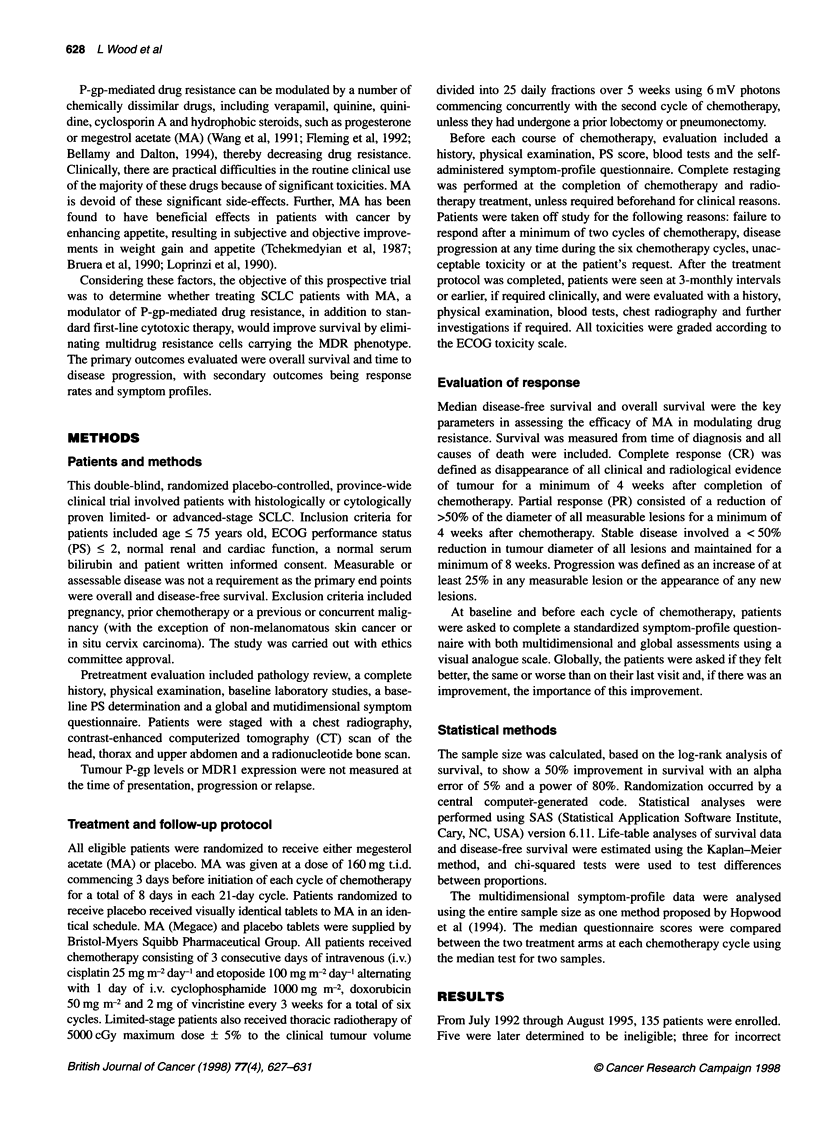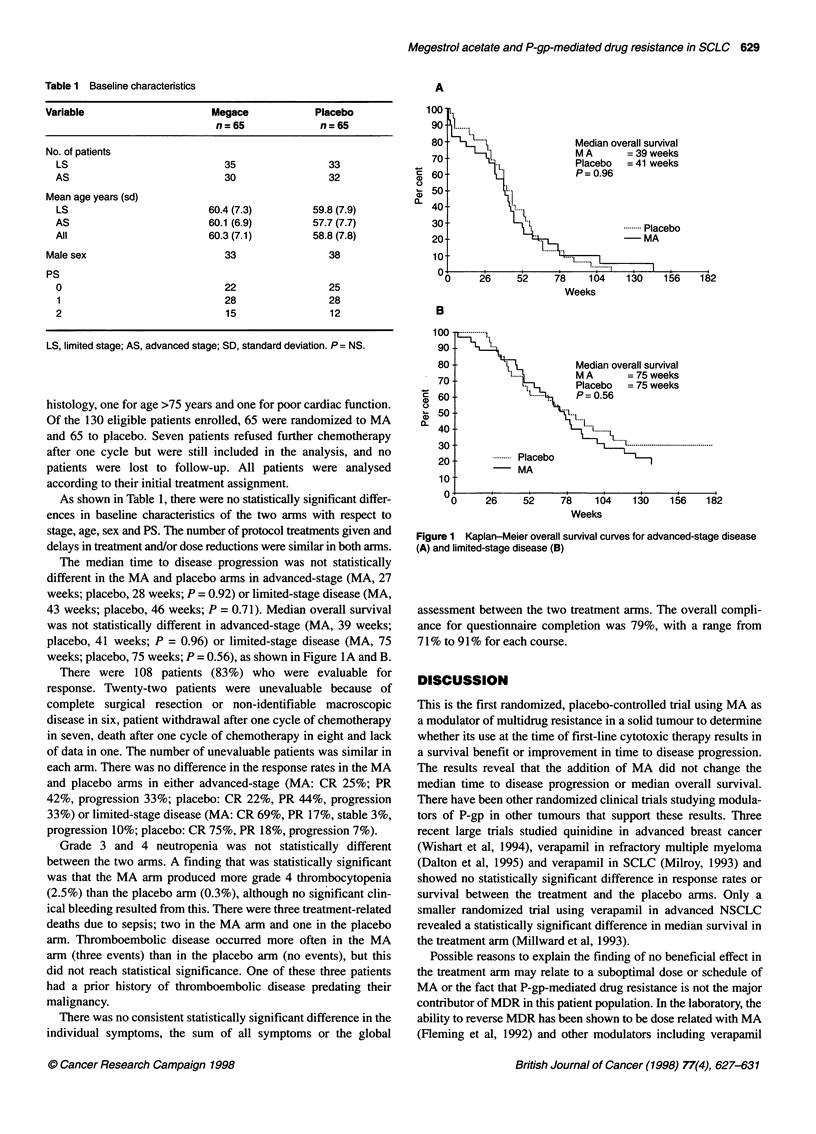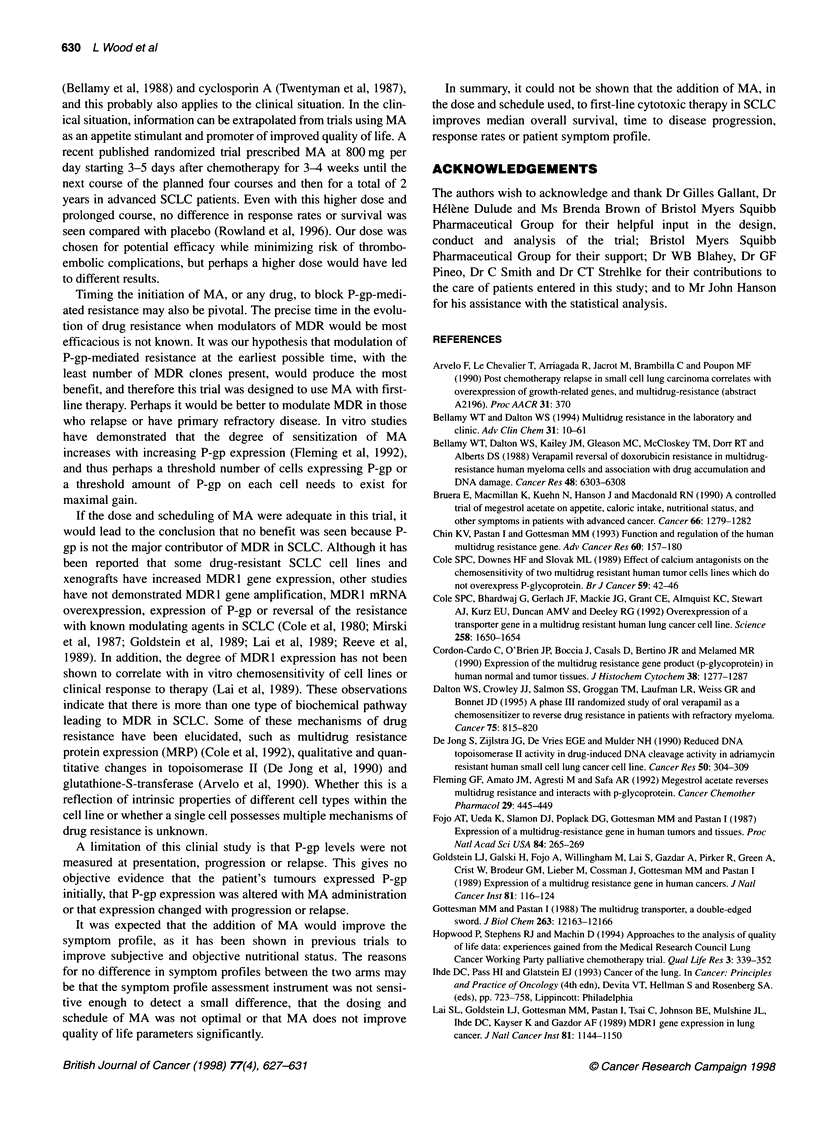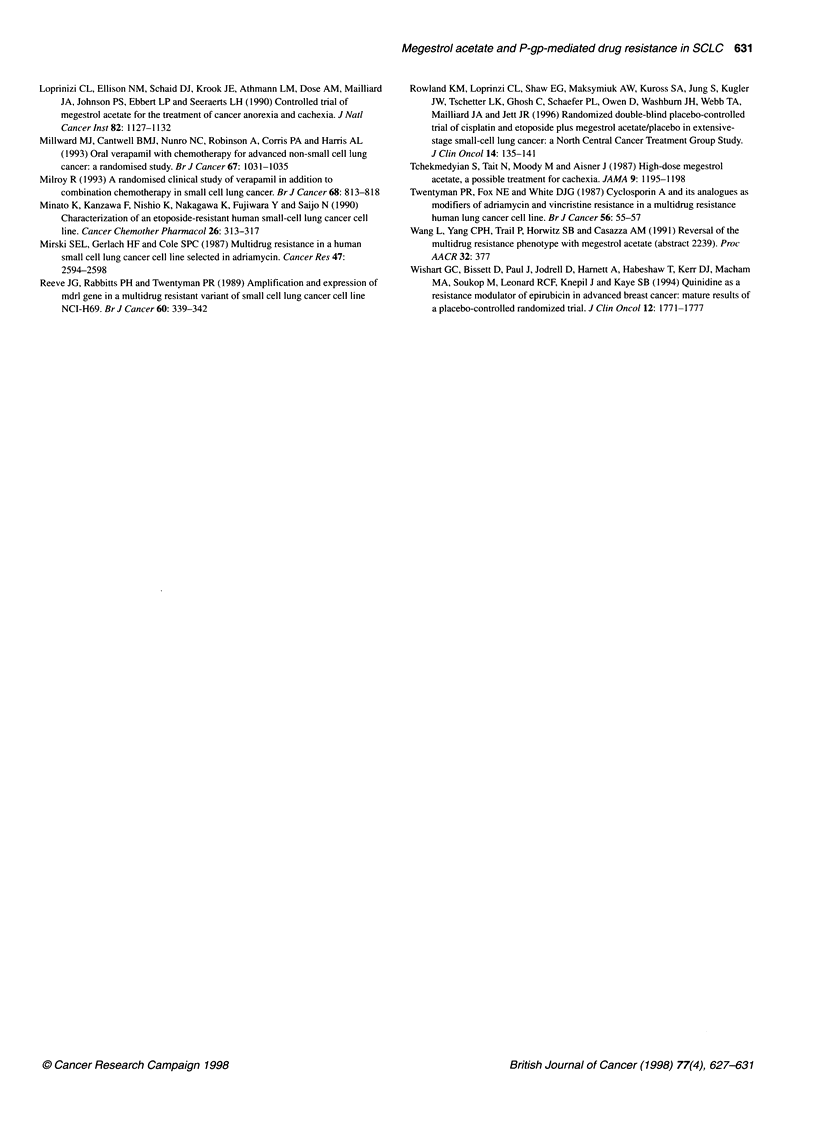# Results of a phase III, double-blind, placebo-controlled trial of megestrol acetate modulation of P-glycoprotein-mediated drug resistance in the first-line management of small-cell lung carcinoma.

**DOI:** 10.1038/bjc.1998.100

**Published:** 1998-02

**Authors:** L. Wood, M. Palmer, J. Hewitt, R. Urtasun, E. Bruera, E. Rapp, J. F. Thaell

**Affiliations:** Cross Cancer Institute, Edmonton, AB, Canada.

## Abstract

The objective of this study was to determine if the addition of megestrol acetate (MA), a modulator of P-glycoprotein-mediated drug resistance, to first-line cytotoxic therapy in patients with limited and advanced stage small-cell lung cancer (SCLC) would improve median time to disease progression and median overall survival. Secondary outcomes evaluated were response rates and patient symptom profile. Between 1992 and 1995, 130 eligible patients were randomized in a double-blind fashion to receive standard first-line therapy consisting of alternating courses of cyclophosphamide/doxorubicin/vincristine and etoposide/cisplatin (and thoracic radiotherapy for limited stage patients), along with either placebo or MA 160 mg t.i.d. for 8 days commencing 3 days before initiation of each cycle of chemotherapy. Treatment was continued for a maximum of six cycles. A total of 130 eligible patients were randomized, 65 to each arm. Fifty-two per cent of patients had limited disease and 48% had advanced disease. The median time to disease progression in limited stage disease was 46 weeks in the placebo arm and 43 weeks in the MA arm (P = 0.71) and in advanced stage disease was 28 weeks in the placebo arm and 27 weeks in the MA arm (P = 0.92). The median overall survival in limited stage disease was 75 weeks in the placebo arm and 75 weeks in the MA arm (P = 0.56) and in advanced stage disease was 41 weeks in the placebo arm and 39 weeks in the MA arm (P = 0.96). There was no consistent statistical difference in response rates or patient symptom profiles between the two treatment arms. The addition of MA, in the dose and schedule used, to standard first-line cytotoxic therapy in SCLC did not result in a significant improvement in response rates, symptom profile, median time to disease progression or overall survival.


British Journal of Cancer (1998) 77(4), 627–631
© 1998 Cancer Research Campaign

### Results of a phase III, double-blind, placebo-controlled trial of megestrol acetate modulation of P-glycoprotein-mediated drug resistance in the first-line management of small-cell lung carcinoma

**L Wood[1], M Palmer[1], J Hewitt[1], R Urtasun[1], E Bruera[1], E Rapp[2] and JF Thaell[3]**

[1]Cross Cancer Institute, 11560 University Avenue, Edmonton, AB, T6G 1Z2, Canada; [2]Tom Baker Cancer Center, 1331 – 29th Street NW, Calgary AB, T2N 4N2, Canada; [3]Calgary General Hospital, 841 Centre Avenue East, Calgary, AB, T2E 0A1, Canada

**Summary** The objective of this study was to determine if the addition of megestrol acetate (MA), a modulator of P-glycoprotein-mediated drug resistance, to first-line cytotoxic therapy in patients with limited and advanced stage small-cell lung cancer (SCLC) would improve median time to disease progression and median overall survival. Secondary outcomes evaluated were response rates and patient symptom profile. Between 1992 and 1995, 130 eligible patients were randomized in a double-blind fashion to receive standard first-line therapy consisting of alternating courses of cyclophosphamide/doxorubicin/vincristine and etoposide/cisplatin (and thoracic radiotherapy for limited stage patients), along with either placebo or MA 160 mg t.i.d. for 8 days commencing 3 days before initiation of each cycle of chemotherapy. Treatment was continued for a maximum of six cycles. A total of 130 eligible patients were randomized, 65 to each arm. Fifty-two per cent of patients had limited disease and 48% had advanced disease. The median time to disease progression in limited stage disease was 46 weeks in the placebo arm and 43 weeks in the MA arm ($P = 0.71$) and in advanced stage disease was 28 weeks in the placebo arm and 27 weeks in the MA arm ($P = 0.92$). The median overall survival in limited stage disease was 75 weeks in the placebo arm and 75 weeks in the MA arm ($P = 0.56$) and in advanced stage disease was 41 weeks in the placebo arm and 39 weeks in the MA arm ($P = 0.96$). There was no consistent statistical difference in response rates or patient symptom profiles between the two treatment arms. The addition of MA, in the dose and schedule used, to standard first-line cytotoxic therapy in SCLC did not result in a significant improvement in response rates, symptom profile, median time to disease progression or overall survival.

**Keywords:** P-glycoprotein; megestrol acetate; small-cell lung cancer; drug resistance

Small-cell lung cancer (SCLC) represents approximately 25% of all bronchogenic carcinoma and, untreated, has a median survival of 12 weeks in limited-stage disease and 5 weeks in advanced-stage disease. Cytotoxic chemotherapy has been shown to produce high initial overall response rates of 75–95% with corresponding improvements in median survival of 12–16 months in limited-stage disease and 7–11 months in advanced-stage disease. However, almost inevitably, the disease relapses and ultimately becomes resistant to further chemotherapy (Ihde et al, 1993, page 723). Resistance to cytotoxic chemotherapy of at least one clone at diagnosis is thought to be the main reason for failure of chemotherapy to maintain the initial response and failure to offer further survival improvement. Thus, a potential strategy for improving overall survival would be to modulate drug resistance.

There are several recognized mechanisms of drug resistance and multidrug resistance (MDR) in malignancies. One mechanism of MDR correlates with an overexpression of P-glycoprotein (P-gp), a 170-kDa transmembrance protein encoded by the MDR1 gene, which acts as an energy-dependent efflux pump to decrease intracellular drug accumulation (Gottesman and Pastan, 1988; Bellamy and Dalton, 1994).

*Received 25 February 1997*
*Revised 19 June 1997*
*Accepted 26 June 1997*

*Correspondence to:* L Wood, Department of Medical Oncology, Cross Cancer Institute, 11560 University Avenue, Edmonton, AB, T6G 1Y9, Canada

P-gp is found in many normal human tissues, including the liver, kidney and colon. These tissues predominantly involve cells lining the luminal space, suggesting a physiological role as a normal transporter for toxic, naturally occurring substances. MDR1 gene expression and P-gp expression has also been reported in a number of solid tumours that are generally regarded as being resistant to primary chemotherapy, including renal cell, colorectal, hepatocellular, adrenal, pancreatic carcinomas and sarcomas (Fojo et al, 1987; Cordon-Cardo et al, 1990; Goldstein et al, 1990). There are also tumours and cell lines initially sensitive to primary chemotherapy that demonstrate undetectable or low levels of P-gp or MDR1 mRNA but on relapse or resistance to chemotherapy express increased levels of P-gp or MDR1 RNA. These malignancies include leukaemias and lymphomas, breast cancer, neuroblastomas, ovarian carcinomas, as well as SCLC (Reeve et al, 1989; Goldstein et al, 1989; Minato et al, 1990; Chin et al, 1993). This is consistent with the concept that malignant cells expressing P-gp at the outset have a selective growth advantage during exposure to cytotoxic drugs. P-gp expression is associated with resistance to a number of natural and semisynthetic cytotoxic drugs in vitro including the anthracyclines, mitomycin C, vinca alkaloids, epipodophyllotoxins and actinomycin D (Chin et al, 1993).

P-gp-mediated drug resistance can be modulated by a number of chemically dissimilar drugs, including verapamil, quinine, quinidine, cyclosporin A and hydrophobic steroids, such as progesterone or megestrol acetate (MA) (Wang et al, 1991; Fleming et al, 1992; Bellamy and Dalton, 1994), thereby decreasing drug resistance. Clinically, there are practical difficulties in the routine clinical use of the majority of these drugs because of significant toxicities. MA is devoid of these significant side-effects. Further, MA has been found to have beneficial effects in patients with cancer by enhancing appetite, resulting in subjective and objective improvements in weight gain and appetite (Tchekmedyian et al, 1987; Bruera et al, 1990; Loprinzi et al, 1990).

Considering these factors, the objective of this prospective trial was to determine whether treating SCLC patients with MA, a modulator of P-gp-mediated drug resistance, in addition to standard first-line cytotoxic therapy, would improve survival by eliminating multidrug resistance cells carrying the MDR phenotype. The primary outcomes evaluated were overall survival and time to disease progression, with secondary outcomes being response rates and symptom profiles.

### METHODS

### Patients and methods

This double-blind, randomized placebo-controlled, province-wide clinical trial involved patients with histologically or cytologically proven limited- or advanced-stage SCLC. Inclusion criteria for patients included age ≤ 75 years old, ECOG performance status (PS) ≤ 2, normal renal and cardiac function, a normal serum bilirubin and patient written informed consent. Measurable or assessable disease was not a requirement as the primary end points were overall and disease-free survival. Exclusion criteria included pregnancy, prior chemotherapy or a previous or concurrent malignancy (with the exception of non-melanomatous skin cancer or in situ cervix carcinoma). The study was carried out with ethics committee approval.

Pretreatment evaluation included pathology review, a complete history, physical examination, baseline laboratory studies, a baseline PS determination and a global and mutidimensional symptom questionnaire. Patients were staged with a chest radiography, contrast-enhanced computerized tomography (CT) scan of the head, thorax and upper abdomen and a radionucleotide bone scan.

Tumour P-gp levels or MDR1 expression were not measured at the time of presentation, progression or relapse.

### Treatment and follow-up protocol

All eligible patients were randomized to receive either megesterol acetate (MA) or placebo. MA was given at a dose of 160 mg t.i.d. commencing 3 days before initiation of each cycle of chemotherapy for a total of 8 days in each 21-day cycle. Patients randomized to receive placebo received visually identical tablets to MA in an identical schedule. MA (Megace) and placebo tablets were supplied by Bristol-Myers Squibb Pharmaceutical Group. All patients received chemotherapy consisting of 3 consecutive days of intravenous (i.v.) cisplatin 25 mg $m^{-2}$ $day^{-1}$ and etoposide 100 mg $m^{-2}$ $day^{-1}$ alternating with 1 day of i.v. cyclophosphamide 1000 mg $m^{-2}$, doxorubicin 50 mg $m^{-2}$ and 2 mg of vincristine every 3 weeks for a total of six cycles. Limited-stage patients also received thoracic radiotherapy of 5000 cGy maximum dose ± 5% to the clinical tumour volume divided into 25 daily fractions over 5 weeks using 6 mV photons commencing concurrently with the second cycle of chemotherapy, unless they had undergone a prior lobectomy or pneumonectomy.

Before each course of chemotherapy, evaluation included a history, physical examination, PS score, blood tests and the self-administered symptom-profile questionnaire. Complete restaging was performed at the completion of chemotherapy and radiotherapy treatment, unless required beforehand for clinical reasons. Patients were taken off study for the following reasons: failure to respond after a minimum of two cycles of chemotherapy, disease progression at any time during the six chemotherapy cycles, unacceptable toxicity or at the patient's request. After the treatment protocol was completed, patients were seen at 3-monthly intervals or earlier, if required clinically, and were evaluated with a history, physical examination, blood tests, chest radiography and further investigations if required. All toxicities were graded according to the ECOG toxicity scale.

### Evaluation of response

Median disease-free survival and overall survival were the key parameters in assessing the efficacy of MA in modulating drug resistance. Survival was measured from time of diagnosis and all causes of death were included. Complete response (CR) was defined as disappearance of all clinical and radiological evidence of tumour for a minimum of 4 weeks after completion of chemotherapy. Partial response (PR) consisted of a reduction of >50% of the diameter of all measurable lesions for a minimum of 4 weeks after chemotherapy. Stable disease involved a < 50% reduction in tumour diameter of all lesions and maintained for a minimum of 8 weeks. Progression was defined as an increase of at least 25% in any measurable lesion or the appearance of any new lesions.

At baseline and before each cycle of chemotherapy, patients were asked to complete a standardized symptom-profile questionnaire with both multidimensional and global assessments using a visual analogue scale. Globally, the patients were asked if they felt better, the same or worse than on their last visit and, if there was an improvement, the importance of this improvement.

### Statistical methods

The sample size was calculated, based on the log-rank analysis of survival, to show a 50% improvement in survival with an alpha error of 5% and a power of 80%. Randomization occurred by a central computer-generated code. Statistical analyses were performed using SAS (Statistical Application Software Institute, Cary, NC, USA) version 6.11. Life-table analyses of survival data and disease-free survival were estimated using the Kaplan–Meier method, and chi-squared tests were used to test differences between proportions.

The multidimensional symptom-profile data were analysed using the entire sample size as one method proposed by Hopwood et al (1994). The median questionnaire scores were compared between the two treatment arms at each chemotherapy cycle using the median test for two samples.

### RESULTS

From July 1992 through August 1995, 135 patients were enrolled. Five were later determined to be ineligible; three for incorrect

© Cancer Research Campaign 1998

**Table 1** Baseline characteristics

| Variable | Megace $n = 65$ | Placebo $n = 65$ |
|---|---|---|
| No. of patients | | |
| LS | 35 | 33 |
| AS | 30 | 32 |
| Mean age years (sd) | | |
| LS | 60.4 (7.3) | 59.8 (7.9) |
| AS | 60.1 (6.9) | 57.7 (7.7) |
| All | 60.3 (7.1) | 58.8 (7.8) |
| Male sex | 33 | 38 |
| PS | | |
| 0 | 22 | 25 |
| 1 | 28 | 28 |
| 2 | 15 | 12 |

LS, limited stage; AS, advanced stage; SD, standard deviation. $P$ = NS.

histology, one for age >75 years and one for poor cardiac function. Of the 130 eligible patients enrolled, 65 were randomized to MA and 65 to placebo. Seven patients refused further chemotherapy after one cycle but were still included in the analysis, and no patients were lost to follow-up. All patients were analysed according to their initial treatment assignment.

As shown in Table 1, there were no statistically significant differences in baseline characteristics of the two arms with respect to stage, age, sex and PS. The number of protocol treatments given and delays in treatment and/or dose reductions were similar in both arms.

The median time to disease progression was not statistically different in the MA and placebo arms in advanced-stage (MA, 27 weeks; placebo, 28 weeks; $P = 0.92$) or limited-stage disease (MA, 43 weeks; placebo, 46 weeks; $P = 0.71$). Median overall survival was not statistically different in advanced-stage (MA, 39 weeks; placebo, 41 weeks; $P = 0.96$) or limited-stage disease (MA, 75 weeks; placebo, 75 weeks; $P = 0.56$), as shown in Figure 1A and B.

There were 108 patients (83%) who were evaluable for response. Twenty-two patients were unevaluable because of complete surgical resection or non-identifiable macroscopic disease in six, patient withdrawal after one cycle of chemotherapy in seven, death after one cycle of chemotherapy in eight and lack of data in one. The number of unevaluable patients was similar in each arm. There was no difference in the response rates in the MA and placebo arms in either advanced-stage (MA: CR 25%; PR 42%, progression 33%; placebo: CR 22%, PR 44%, progression 33%) or limited-stage disease (MA: CR 69%, PR 17%, stable 3%, progression 10%; placebo: CR 75%, PR 18%, progression 7%).

Grade 3 and 4 neutropenia was not statistically different between the two arms. A finding that was statistically significant was that the MA arm produced more grade 4 thrombocytopenia (2.5%) than the placebo arm (0.3%), although no significant clinical bleeding resulted from this. There were three treatment-related deaths due to sepsis; two in the MA arm and one in the placebo arm. Thromboembolic disease occurred more often in the MA arm (three events) than in the placebo arm (no events), but this did not reach statistical significance. One of these three patients had a prior history of thromboembolic disease predating their malignancy.

There was no consistent statistically significant difference in the individual symptoms, the sum of all symptoms or the global assessment between the two treatment arms. The overall compliance for questionnaire completion was 79%, with a range from 71% to 91% for each course.

**Figure 1** Kaplan–Meier overall survival curves for advanced-stage disease (**A**) and limited-stage disease (**B**)

### DISCUSSION

This is the first randomized, placebo-controlled trial using MA as a modulator of multidrug resistance in a solid tumour to determine whether its use at the time of first-line cytotoxic therapy results in a survival benefit or improvement in time to disease progression. The results reveal that the addition of MA did not change the median time to disease progression or median overall survival. There have been other randomized clinical trials studying modulators of P-gp in other tumours that support these results. Three recent large trials studied quinidine in advanced breast cancer (Wishart et al, 1994), verapamil in refractory multiple myeloma (Dalton et al, 1995) and verapamil in SCLC (Milroy, 1993) and showed no statistically significant difference in response rates or survival between the treatment and the placebo arms. Only a smaller randomized trial using verapamil in advanced NSCLC revealed a statistically significant difference in median survival in the treatment arm (Millward et al, 1993).

Possible reasons to explain the finding of no beneficial effect in the treatment arm may relate to a suboptimal dose or schedule of MA or the fact that P-gp-mediated drug resistance is not the major contributor of MDR in this patient population. In the laboratory, the ability to reverse MDR has been shown to be dose related with MA (Fleming et al, 1992) and other modulators including verapamil

© Cancer Research Campaign 1998

(Bellamy et al, 1988) and cyclosporin A (Twentyman et al, 1987), and this probably also applies to the clinical situation. In the clinical situation, information can be extrapolated from trials using MA as an appetite stimulant and promoter of improved quality of life. A recent published randomized trial prescribed MA at 800 mg per day starting 3–5 days after chemotherapy for 3–4 weeks until the next course of the planned four courses and then for a total of 2 years in advanced SCLC patients. Even with this higher dose and prolonged course, no difference in response rates or survival was seen compared with placebo (Rowland et al, 1996). Our dose was chosen for potential efficacy while minimizing risk of thromboembolic complications, but perhaps a higher dose would have led to different results.

Timing the initiation of MA, or any drug, to block P-gp-mediated resistance may also be pivotal. The precise time in the evolution of drug resistance when modulators of MDR would be most efficacious is not known. It was our hypothesis that modulation of P-gp-mediated resistance at the earliest possible time, with the least number of MDR clones present, would produce the most benefit, and therefore this trial was designed to use MA with first-line therapy. Perhaps it would be better to modulate MDR in those who relapse or have primary refractory disease. In vitro studies have demonstrated that the degree of sensitization of MA increases with increasing P-gp expression (Fleming et al, 1992), and thus perhaps a threshold number of cells expressing P-gp or a threshold amount of P-gp on each cell needs to exist for maximal gain.

If the dose and scheduling of MA were adequate in this trial, it would lead to the conclusion that no benefit was seen because P-gp is not the major contributor of MDR in SCLC. Although it has been reported that some drug-resistant SCLC cell lines and xenografts have increased MDR1 gene expression, other studies have not demonstrated MDR1 gene amplification, MDR1 mRNA overexpression, expression of P-gp or reversal of the resistance with known modulating agents in SCLC (Cole et al, 1980; Mirski et al, 1987; Goldstein et al, 1989; Lai et al, 1989; Reeve et al, 1989). In addition, the degree of MDR1 expression has not been shown to correlate with in vitro chemosensitivity of cell lines or clinical response to therapy (Lai et al, 1989). These observations indicate that there is more than one type of biochemical pathway leading to MDR in SCLC. Some of these mechanisms of drug resistance have been elucidated, such as multidrug resistance protein expression (MRP) (Cole et al, 1992), qualitative and quantitative changes in topoisomerase II (De Jong et al, 1990) and glutathione-S-transferase (Arvelo et al, 1990). Whether this is a reflection of intrinsic properties of different cell types within the cell line or whether a single cell possesses multiple mechanisms of drug resistance is unknown.

A limitation of this clinial study is that P-gp levels were not measured at presentation, progression or relapse. This gives no objective evidence that the patient's tumours expressed P-gp initially, that P-gp expression was altered with MA administration or that expression changed with progression or relapse.

It was expected that the addition of MA would improve the symptom profile, as it has been shown in previous trials to improve subjective and objective nutritional status. The reasons for no difference in symptom profiles between the two arms may be that the symptom profile assessment instrument was not sensitive enough to detect a small difference, that the dosing and schedule of MA was not optimal or that MA does not improve quality of life parameters significantly.

In summary, it could not be shown that the addition of MA, in the dose and schedule used, to first-line cytotoxic therapy in SCLC improves median overall survival, time to disease progression, response rates or patient symptom profile.

### ACKNOWLEDGEMENTS

The authors wish to acknowledge and thank Dr Gilles Gallant, Dr Hélène Dulude and Ms Brenda Brown of Bristol Myers Squibb Pharmaceutical Group for their helpful input in the design, conduct and analysis of the trial; Bristol Myers Squibb Pharmaceutical Group for their support; Dr WB Blahey, Dr GF Pineo, Dr C Smith and Dr CT Strehlke for their contributions to the care of patients entered in this study; and to Mr John Hanson for his assistance with the statistical analysis.

### REFERENCES

Arvelo F, Le Chevalier T, Arriagada R, Jacrot M, Brambilla C and Poupon MF (1990) Post chemotherapy relapse in small cell lung carcinoma correlates with overexpression of growth-related genes, and multidrug-resistance (abstract A2196). *Proc AACR* **31**: 370

Bellamy WT and Dalton WS (1994) Multidrug resistance in the laboratory and clinic. *Adv Clin Chem* **31**: 10–61

Bellamy WT, Dalton WS, Kailey JM, Gleason MC, McCloskey TM, Dorr RT and Alberts DS (1988) Verapamil reversal of doxorubicin resistance in multidrug-resistance human myeloma cells and association with drug accumulation and DNA damage. *Cancer Res* **48**: 6303–6308

Bruera E, Macmillan K, Kuehn N, Hanson J and Macdonald RN (1990) A controlled trial of megestrol acetate on appetite, caloric intake, nutritional status, and other symptoms in patients with advanced cancer. *Cancer* **66**: 1279–1282

Chin KV, Pastan I and Gottesman MM (1993) Function and regulation of the human multidrug resistance gene. *Adv Cancer Res* **60**: 157–180

Cole SPC, Downes HF and Slovak ML (1989) Effect of calcium antagonists on the chemosensitivity of two multidrug resistant human tumor cells lines which do not overexpress P-glycoprotein. *Br J Cancer* **59**: 42–46

Cole SPC, Bhardwaj G, Gerlach JF, Mackie JG, Grant CE, Almquist KC, Stewart AJ, Kurz EU, Duncan AMV and Deeley RG (1992) Overexpression of a transporter gene in a multidrug resistant human lung cancer cell line. *Science* **258**: 1650–1654

Cordon-Cardo C, O'Brien JP, Boccia J, Casals D, Bertino JR and Melamed MR (1990) Expression of the multidrug resistance gene product (p-glycoprotein) in human normal and tumor tissues. *J Histochem Cytochem* **38**: 1277–1287

Dalton WS, Crowley JJ, Salmon SS, Groggan TM, Laufman LR, Weiss GR and Bonnet JD (1995) A phase III randomized study of oral verapamil as a chemosensitizer to reverse drug resistance in patients with refractory myeloma. *Cancer* **75**: 815–820

De Jong S, Zijlstra JG, De Vries EGE and Mulder NH (1990) Reduced DNA topoisomerase II activity in drug-induced DNA cleavage activity in adriamycin resistant human small cell lung cancer cell line. *Cancer Res* **50**: 304–309

Fleming GF, Amato JM, Agresti M and Safa AR (1992) Megestrol acetate reverses multidrug resistance and interacts with p-glycoprotein. *Cancer Chemother Pharmacol* **29**: 445–449

Fojo AT, Ueda K, Slamon DJ, Poplack DG, Gottesman MM and Pastan I (1987) Expression of a multidrug-resistance gene in human tumors and tissues. *Proc Natl Acad Sci USA* **84**: 265–269

Goldstein LJ, Galski H, Fojo A, Willingham M, Lai S, Gazdar A, Pirker R, Green A, Crist W, Brodeur GM, Lieber M, Cossman J, Gottesman MM and Pastan I (1989) Expression of a multidrug resistance gene in human cancers. *J Natl Cancer Inst* **81**: 116–124

Gottesman MM and Pastan I (1988) The multidrug transporter, a double-edged sword. *J Biol Chem* **263**: 12163–12166

Hopwood P, Stephens RJ and Machin D (1994) Approaches to the analysis of quality of life data: experiences gained from the Medical Research Council Lung Cancer Working Party palliative chemotherapy trial. *Qual Life Res* **3**: 339–352

Ihde DC, Pass HI and Glatstein EJ (1993) Cancer of the lung. In *Cancer: Principles and Practice of Oncology* (4th edn), Devita VT, Hellman S and Rosenberg SA. (eds), pp. 723–758, Lippincott: Philadelphia

Lai SL, Goldstein LJ, Gottesman MM, Pastan I, Tsai C, Johnson BE, Mulshine JL, Ihde DC, Kayser K and Gazdor AF (1989) MDR1 gene expression in lung cancer. *J Natl Cancer Inst* **81**: 1144–1150

© Cancer Research Campaign 1998

Loprinizi CL, Ellison NM, Schaid DJ, Krook JE, Athmann LM, Dose AM, Mailliard JA, Johnson PS, Ebbert LP and Seeraerts LH (1990) Controlled trial of megestrol acetate for the treatment of cancer anorexia and cachexia. *J Natl Cancer Inst* **82**: 1127–1132

Millward MJ, Cantwell BMJ, Nunro NC, Robinson A, Corris PA and Harris AL (1993) Oral verapamil with chemotherapy for advanced non-small cell lung cancer: a randomised study. *Br J Cancer* **67**: 1031–1035

Milroy R (1993) A randomised clinical study of verapamil in addition to combination chemotherapy in small cell lung cancer. *Br J Cancer* **68**: 813–818

Minato K, Kanzawa F, Nishio K, Nakagawa K, Fujiwara Y and Saijo N (1990) Characterization of an etoposide-resistant human small-cell lung cancer cell line. *Cancer Chemother Pharmacol* **26**: 313–317

Mirski SEL, Gerlach HF and Cole SPC (1987) Multidrug resistance in a human small cell lung cancer cell line selected in adriamycin. *Cancer Res* **47**: 2594–2598

Reeve JG, Rabbitts PH and Twentyman PR (1989) Amplification and expression of mdrl gene in a multidrug resistant variant of small cell lung cancer cell line NCI-H69. *Br J Cancer* **60**: 339–342

Rowland KM, Loprinzi CL, Shaw EG, Maksymiuk AW, Kuross SA, Jung S, Kugler JW, Tschetter LK, Ghosh C, Schaefer PL, Owen D, Washburn JH, Webb TA, Mailliard JA and Jett JR (1996) Randomized double-blind placebo-controlled trial of cisplatin and etoposide plus megestrol acetate/placebo in extensive-stage small-cell lung cancer: a North Central Cancer Treatment Group Study. *J Clin Oncol* **14**: 135–141

Tchekmedyian S, Tait N, Moody M and Aisner J (1987) High-dose megestrol acetate, a possible treatment for cachexia. *JAMA* **9**: 1195–1198

Twentyman PR, Fox NE and White DJG (1987) Cyclosporin A and its analogues as modifiers of adriamycin and vincristine resistance in a multidrug resistance human lung cancer cell line. *Br J Cancer* **56**: 55–57

Wang L, Yang CPH, Trail P, Horwitz SB and Casazza AM (1991) Reversal of the multidrug resistance phenotype with megestrol acetate (abstract 2239). *Proc AACR* **32**: 377

Wishart GC, Bissett D, Paul J, Jodrell D, Harnett A, Habeshaw T, Kerr DJ, Macham MA, Soukop M, Leonard RCF, Knepil J and Kaye SB (1994) Quinidine as a resistance modulator of epirubicin in advanced breast cancer: mature results of a placebo-controlled randomized trial. *J Clin Oncol* **12**: 1771–1777

© Cancer Research Campaign 1998